# Neurogenic bowel dysfunction

**DOI:** 10.12688/f1000research.20529.1

**Published:** 2019-10-28

**Authors:** Anton Emmanuel

**Affiliations:** 1 GI Physiology Unit, University College London Hospital, Euston Road, London, WC1E 6DB, UK

**Keywords:** Neurogenic Bladder, Bowel Dysfunction, Constipation, Faecal incontinence

## Abstract

The symptoms of neurogenic bowel dysfunction (NBD) comprise constipation and fecal incontinence. These have a major impact on quality of life and dignity. Bowel symptoms occur in the majority of patients with chronic neurological diseases like multiple sclerosis, spinal cord injury, and Parkinson’s disease. Management relies on obtaining a careful bowel history, including assessment of bowel function prior to the onset of neurological symptoms. Objective measures of NBD are available and important in terms of monitoring response for what are often intensely personal and difficult-to-elicit symptoms. Conservative management begins by establishing an effective and regular bowel regime by optimizing diet and laxative use. If this is insufficient, as seen in about half of patients, transanal irrigation has been shown to reduce NBD symptoms and improve quality of life. Failing that, there are more invasive surgical options available. This review aims to provide practical guidance for the clinician who encounters these patients, focusing on a stepwise approach to assessment, interventions, and monitoring.

## Introduction

The number of individuals vulnerable to bowel and bladder dysfunction is ever-increasing. Spinal cord injury (SCI), both traumatic and non-traumatic, has an estimated prevalence of over 2.5 million worldwide ^1^ and an incidence of 15 per million in the UK ^2^. Similarly, multiple sclerosis (MS; the commonest disabling neurological disease of young adults) and Parkinson’s disease (the second commonest neurodegenerative disease) affect over 1.5 million and 3 million people, respectively ^3^.

Amongst those suffering from central nervous system injury or disease, bowel symptoms are experienced commonly ^4, 5^. Of those with SCI, up to 95% report constipation ^6^ and 75% have experienced episodes of fecal incontinence ^7^. Two-thirds of individuals with MS experience constipation and/or fecal incontinence ^5^, and in spina bifida patients only 32% report normal bowel function ^8^. Constipation affects over 25–63% of those with Parkinson’s disease (depending on definition used) ^9^. Stroke may also result in neurogenic bowel dysfunction (NBD), with chronic fecal incontinence occurring in 15% of patients ^10^.

The symptoms of NBD have a substantial negative impact on quality of life, social integration, and personal independence ^11^. Only 6% of SCI patients require no intervention to support their bowel function ^12^. As many as 65% need to employ intrusive options such as digital stimulation or evacuation of the anorectum ^7^, and one-third require assistance with bowel care ^12^. In 22% of individuals with SCI, bowel management takes up to an hour on every occasion, and in 14% it takes over 60 minutes ^7^. The consequences of all this are loss of independence and dignity, embarrassment, anxiety, depression, social isolation, and loss of sexual relationships ^11, 13^. In fact, the burden of NBD is so great that SCI patients report bowel dysfunction as more problematic than any of bladder dysfunction, sexual dysfunction, pain, fatigue, or perception of body image ^6^.

## Assessment

A GI history should be obtained from the patient and any carers they may have. Details of bowel habit prior to injury or neurological disease onset should be explored. This is especially relevant in Parkinson’s disease patients, where bowel dysfunction can begin decades before neurological symptoms ^14^. A bowel diary can be especially helpful to quantify symptoms over a one- or two-week period as well as helping to identify any factors that influence bowel function.

Current symptoms should be thoroughly assessed, including frequency of bowel motions, stool consistency (the Bristol Stool Form scale can be helpful), episodes of fecal or flatus incontinence or urgency, maneuvers required for bowel management (digital anorectal stimulation or digital evacuation), time spent toileting, episodes of fecal impaction, laxative/anti-diarrheal usage, need for pads/plugs ^11, 14, 15^, and limitations on quality of life engendered by bowel symptoms.

The presence of associated morbidity should also be considered. For NBD, this can include urinary tract infections (UTIs), hemorrhoids, abdominal pain, rectal bleeding and prolapse, anal fissures, and autonomic dysreflexia ^16– 18^. Autonomic dysreflexia most commonly occurs in individuals with incomplete SCI and may present as feelings of general uneasiness, headache, and perspiration that occur during defecation ^19^. In children, fussiness and irritability may be more suggestive ^20^. Frequency and content of meals should also be gauged at this stage ^11^.

Past medical history must include any co-existing GI or anal sphincter dysfunction. Functional bowel disorders such as irritable bowel syndrome and anal sphincter lesions from childbirth are common amongst the general population and may contribute to symptoms of NBD such as fecal incontinence ^21, 22^. This may affect treatment outcomes. Prior histories of pelvic organ prolapses or surgical procedures involving the GI tract (including the perianal region) are also relevant ^14^. Symptoms may deteriorate with time: a longitudinal study suggested that patients require increasingly intrusive treatment options to maintain quality of life ^23^.

Scoring systems may be helpful in quantifying symptoms. Standard instruments such as the Cleveland constipation score and St Mark’s incontinence score may be used depending on dominant symptoms ^24, 25^. A condition-specific NBD score has been developed and is validated in SCI and adolescents/children with spina bifida, but not in Parkinson’s disease ^26– 28^. It has been used in MS ^29^.

Digital rectal examination is a necessary part of assessment and should include evaluation of rectal filling, resting anal tone, and ability to produce a voluntary contraction. This will also provide a crude assessment of anal sensitivity ^14^. Perineal sensitivity may be examined by pinprick ^11^. Formal anorectal physiology studies are not usually required if physical examination is adequate ^11^. Examination should also include looking for complications of chronic constipation, namely anal fissures, complicated hemorrhoids, rectal bleeding, and prolapse.

## Investigations

As with non-NBD patients, appropriate colonic imaging should be performed in the presence of any “red flag” symptoms. Clearly, these are harder to recognize in patients with NBD. In general, any worsening of bowel dysfunction or weight or blood loss signals a need for further investigation ^14^.

More invasive physiological or radiological transit investigations do not have an established place in assessment. Whilst there is some suggestion that electrophysiology may identify neural dysfunction as a cause of anorectal symptoms, this has not been universally shown ^7, 12, 14^. Their use may be appropriate in the presence of co-morbidity such as prior anal surgery, obstetric history, or pelvic organ prolapse ^14^. Otherwise, evidence of slow transit can usually be obtained from history based on an urge frequency of daily or less in the presence of hard stool ^11^.

The most frequently undertaken radiological testing comprises colonic transit studies. Typically, these are undertaken by taking an abdominal X-ray at a fixed time a few days after ingesting radio-opaque markers, and there exist a range of protocols ^30^. In patients with central neurological disease, these characteristically show delayed transit, which may relate to neuropathy secondary to the disease, reduced mobility, or concomitant medication. Anorectal physiology testing involves the assessment of anorectal pressures, sensation, and coordination through minimally invasive manometry and electrophysiology ^31^. These tests are capable of identifying hindgut denervation as part of the disease, indicating severity.

## Treatments

Traditionally, treatment begins with a conservative approach focusing on optimizing the bowel regime and progresses to more invasive measures as needed. Invasive measures can be categorized into irrigation methods, electrical stimulation therapies, and surgical procedures ^11^. There is, however, an increasing recognition that treatment needs to be tailored to the individual’s situation, and some patients may opt for a more interventional approach in the first instance ^15^.

### Conservative bowel regime

The three cardinal aims of bowel care are to ensure toileting occurs in as time efficient a way as possible, to avoid fecal incontinence, and to minimize impairment of quality of life secondary to the bowel management plan ^32^. This can be achieved through the use of diet, bowel routine, medication, and physical techniques. No matter the methods used, patient education and training are central to achieving success ^32^.

Diet should be optimized to suit the predominant symptoms. In patients with a slow bowel, a diet high in fiber is likely to cause bloating and flatulence. These symptoms may be improved by decreasing the amount of insoluble fiber, especially cereals, in the diet. Conversely, for those with accelerated bowel transit, higher levels of fiber in the diet may help to bulk up stools and so decrease the likelihood of soiling. For these patients, products that loosen the stools, such as caffeine, alcohol, and food containing the sweetener sorbitol, should also be used with caution. Whatever the diet content, the most important step in achieving optimal bowel motility is to establish a regular eating pattern ^11^. Fluid intake should also be optimized whilst taking bladder constraints into account ^33^.

Medication history should be reviewed, as drugs such as bladder anticholinergics, opiates, non-steroidal anti-inflammatory drugs (NSAIDs), and antibiotics may all contribute to bowel dysfunction ^11^.

Establishing a routine for bowel care is also vitally important. Patients should attempt to defecate at a scheduled time, either daily or on alternate days. Frequency would depend on the individual’s bowel habit prior to the onset of their neurological disorder. Chances of success may be maximized by scheduling defecation to occur when bowel contractions are strongest: on waking and after a meal/warm drink. Privacy and comfort for the patient are also of crucial importance in creating an environment conducive to successful defecation. Similarly, position during toileting can be used to maximize bowel efficiency. Gravity can be best exploited in a seated position, on a toilet or commode, if this is practical for the patient ^11^.

There are a number of adjuvant techniques to assist bowel evacuation, according to the patient’s hand function and toileting independence. Abdominal massage involves using the heel of the palm to massage the abdomen from left to right in circular movements. The Valsalva maneuver is performed by attempting exhalation against a closed airway (closed glottis or pinched nose) to ensure effective propulsion. Digital rectal stimulation is where a gloved and lubricated finger is inserted into the anus and moved in a circular motion for 20–30 seconds; its aim is to stimulate the recto-colic reflex and therefore a bowel movement. The process can be repeated again 5 minutes later if required and should be used with caution in SCI patients, as it may induce autonomic dysreflexia ^19^. Digital evacuation of stools does not depend on contraction but involves physically removing formed stools present in the rectum using a hooking motion; using a Valsalva maneuver simultaneously may improve efficacy. Suppositories and enemas may also aid in stimulating reflex contraction; they should be used only if stool is present within the rectum on digital rectal examination and should be retained for a minimum of 10 minutes. Available options are glycerin (lubricant), bisacodyl (stimulant), and lecicarbon (carbon dioxide releasing). Anal plugs prevent the leakage of flatus and small volumes of feces in those with passive incontinence and are best tolerated in patients with reduced anal sensation. Suppository inserters, finger extensions, digital stimulators, and perineal cleaners may all help to preserve patient independence when carrying out bowel care. Appropriate toilet adaptations should be employed to optimize patient comfort ^11^.

### Transanal irrigation methods

Transanal irrigation (TAI) assists the evacuation of feces from the bowel by introducing water into the colon and rectum through the anus in order to induce a reflex colorectal voiding. The water is introduced using a single-use device, either a single-use cone or catheter. The choice of cone or catheter depends on patient choice, hand function, and anal sphincter integrity; a cone is preferred if the patient can retain the device *in situ* by hand or sphincter tone whilst instilling the fluid. After the device is removed, the contents of the rectum and some of the more proximal colon is emptied. With regular use, TAI aids the re-establishment of controlled bowel function and allows the patient to control when and where evacuation takes place. In the case of fecal incontinence, effective evacuation of the colon and rectum delays the arrival of new feces for approximately two days, which prevents leakage occurring between irrigations ^34^. Regular emptying of the rectosigmoid region in those suffering from constipation could avert blockages by supporting transport through the whole colon.

A study into the long-term results of TAI found that approximately 60% continue with treatment at long-term follow-up and resulted in lower rates of stoma surgery, UTIs, and episodes of fecal incontinence with improved quality-adjusted life years compared to conservative bowel care ^15^. This was associated with cost savings of £21,768 per patient compared to continuation of standard bowel care ^15^.

### Electrical stimulation

Nerve stimulation via implantation of electrodes is another method that has been explored in some patients who have failed conservative therapy. The approach involves implanting the electrodes onto sacral roots. This sacral nerve stimulation is thought to have an effect on both afferent fibers to the brain and sacral efferents ^11^. A more invasive approach requires a laparotomy to implant a sacral anterior root stimulator. This can be accompanied by a posterior rhizotomy (to prevent autonomic dysreflexia) followed by the placement of electrodes on the efferent sacral roots ^35^. Although these devices are more commonly implanted for bladder control, their positive effects on bowel function have been shown to be significant ^35^. However, the technical complexity, expense, and invasiveness of the methodology means they are little used. Alternate forms of neuromodulation have been studied in patients with NBD but with limited efficacy and wider uptake despite being described many years ago ^36^.

### Surgical antegrade colonic irrigation

Antegrade irrigation through an appendicostomy has been used in children with NBD, particularly those with spina bifida, providing long-term success in over 80% of patients ^37^. Unfortunately, results in adults have been less promising, the main problem being the development of tract stenosis ^38^. Another limitation of this approach is that it can take time for the whole colon to be washed out ^11^.

Alternatively, irrigation may be performed via a percutaneous endoscopic colostomy. In this approach, a tube is placed into the sigmoid colon and used to wash out the distal bowel. Although effective in the majority of patients, the technique can have substantial complications, making it a less useful approach in the long term ^11, 39^.

### Stoma formation

Surgical formation of a stoma is generally considered a last option, since it is invasive and not simply reversible. However, it can be extremely successful for patients with good use of their upper limbs and when fecal incontinence dominates ^11^. Stoma formation is associated with improved quality of life and reduced bowel management time ^40^. Unfortunately, complications (including rectal mucus discharge, diversion colitis, and post-surgical adhesions) may be as high as 37.5% ^32, 41, 42^. Furthermore, laxatives or stoma irrigation may still be necessary, unless a loop ileostomy is performed ^11^. In terms of location, left-sided colostomy may be the most suitable for those requiring fecal diversion due to complicated perianal wounds. However, this approach is associated with poor colonic emptying and so should be for those with good colonic motility. Right-sided colostomy is less likely to cause these problems but results in more liquid stools, increased stoma care requirements, and greater risk of leaks ^43^.

**Figure 1.**
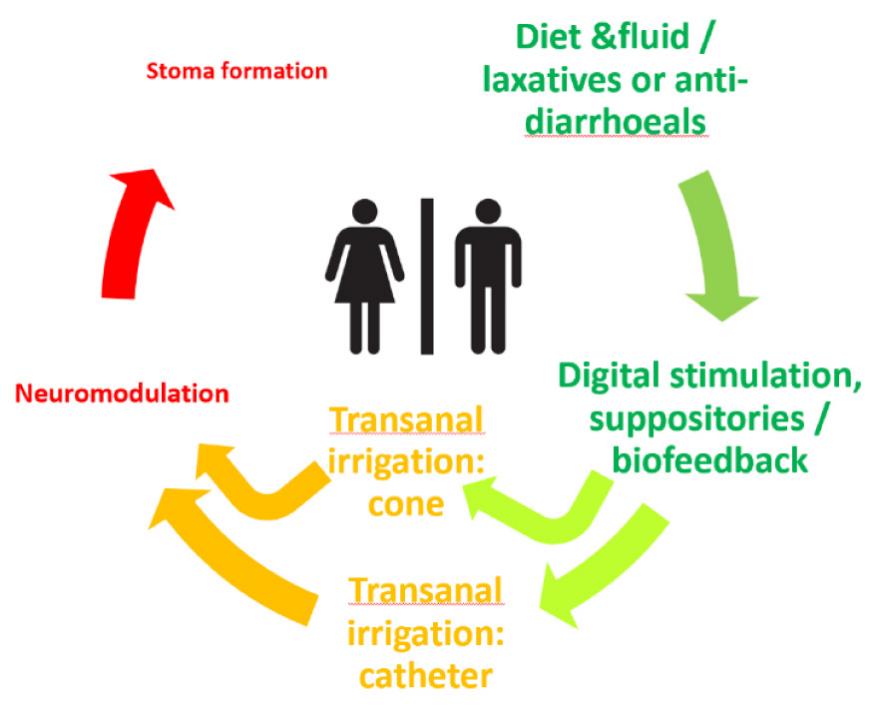
Care options for the management of neurogenic bowel dysfunction. Font size reflects the frequency of the options being used. Color gradation: green = conservative, orange = minimally invasive, red = invasive ^15^.

A treatment hierarchy has been proposed ( Figure 1), an alternative to the traditional “pyramid”, as it reflects the range of options available and their frequency of use rather than indicating an evidence-based patient pathway.

## Summary

Bowel dysfunction is a pathophysiological term that encompasses symptoms that can have an overwhelming effect on a patient’s life. Fecal incontinence and chronic constipation are the symptoms that result, and these have a large impact on the patient’s ability to function in a social or work capacity. Patients who develop these in the context of neurological disease have an especially heavy burden, as the neurodisability aspect has an impact on the presentation and management of these symptoms. Approximately two out of three patients with neurological disease develop bowel symptoms in the course of their illness, and these may deteriorate with time. Those individuals who develop bowel dysfunction have greater rates of hospitalization and health care utilization. Assessment is directed towards understanding the impact of the symptoms on the individual’s quality of life and determining if the resulting dysfunction is associated with a reflexic (upper motor neuron type lesion) or flaccid (lower motor neuron) pathophysiology.

Treatment is directed at avoiding fecal incontinence, minimizing time spent toileting, preventing complications (including UTIs), and optimizing quality of life. Lifestyle interventions, tailoring of the bowel regime, and laxative manipulation are the first-line approaches. These are based on clinical experience in the absence of a strong evidence base. The use of TAI has emerged as a well-studied option with a long-term response rate of over 60%. In refractory cases, or according to patient choice, surgical stoma formation is associated with improved quality of life.
